# Supplementary material for: Occurrence and stability of hetero-hexamer associations formed by β-carboxysome CcmK shell components
Source: PLoS One. 2019 Oct 11;14(10):e0223877. doi: 10.1371/journal.pone.0223877 (PMC6788708; doi:10.1371/journal.pone.0223877)
Supplement: S1 Table — (DOCX) [file pone.0223877.s001.docx]

**S1 Table – Analysis of native-MS and MS-MS data collected on CcmK1/K2 and CcmK3/K4 hetero-hexamers.**

| Sample | Theor.  MW^a^ | Exp.  MW | Stoich. | Deviation^b^ |
| --- | --- | --- | --- | --- |
| K1*/K2* | 13153.0 (K1m)  12185.8 (K2m)  73114.8 (K2h)  78918.0 (K1h) | 12988  12041  73184  **74212**  **75143**  76078  122346  123380  124206 | 1  1  6  1:5  2:4  3:3  4:2  2:8  3:7  4:6 | - 165  -145  -9  +72  +56  +44  +42  +129  +8 |
| K1* + K2* | 13153.0 (K1m)  78918.0 (K1h) | 13012  77694  **78123** | 1  6:0  6:0 | -141  -234  +195 |
| *K4/K3* | 12085.8 (K4m)  11972.8 (K3m)  71836.8 (K3h)  72514.8 (K4h) | 12087  11842  12094  37783  66852  71087  **72334**  72568 | 1  1  1  NA  NA  0:6  5:1  6:0 | +1  -131  +8 (K4)  +35  +57  +46 |

^a^ Theoretical molecular weights calculated from amino acid sequences. The values of theoretical hexamers are also given (indicated by h letter following the isoform name). ^b^ Mass deviations between experimental and theoretical values are indicated. When considering monomers, the deviation is with regard to theoretical molecular weight; In the case of mixed associations, the deviation is with regard to values calculated for such combinations of experimental masses measured for each component as monomer. This is basically to account for typical losses of first methionine residue (131.04 Da) with components tagged at C-terminus.
