# Supplementary material for: Occurrence and stability of hetero-hexamer associations formed by β-carboxysome CcmK shell components
Source: PLoS One. 2019 Oct 11;14(10):e0223877. doi: 10.1371/journal.pone.0223877 (PMC6788708; doi:10.1371/journal.pone.0223877)
Supplement: S3 Table — (DOCX) [file pone.0223877.s003.docx]

**S3 Table – Crystallographic data and refinement statistics**

| Cell parameters (Å, °) | *a* = *b* = 70.68, *c* = 64.79, α = β = 90, γ = 120 | |
| --- | --- | --- |
| Space group | *P*6_3_ | |
| Resolution range (Å) | 61.21 – 1.80 | 1.84 – 1.80 |
| Nb. of observations | 178945 | 10458 |
| Nb. of unique reflections | 17065 | 994 |
| Multiplicity | 10.5 | 10.5 |
| Completeness (%) | 99.6 | 99.7 |
| Rmerge | 0.095 | 1.341 |
| Rmeas | 0.100 | 1.348 |
| Rpim | 0.031 | 0.414 |
| I/σ | 14.1 | 1.9 |
| CC(1/2) | 0.999 | 0.847 |
| Resolution range (Å) | 61.20 – 1.80 |  |
| Nb. of reflections | 17024 |  |
| Completeness | 99.4 |  |
| Nb. of atoms |  |  |
| Protein | 3067 |  |
| Water, ethylene glycol | 157 |  |
| Rfactor | 0.1710 |  |
| Rfree | 0.1924 |  |
| rmsd bond lengths (Å) | 0.006 |  |
| rmsd bond angles (°) | 0.733 |  |
