# Supplementary material for: Occurrence and stability of hetero-hexamer associations formed by β-carboxysome CcmK shell components
Source: PLoS One. 2019 Oct 11;14(10):e0223877. doi: 10.1371/journal.pone.0223877 (PMC6788708; doi:10.1371/journal.pone.0223877)
Supplement: S4 Table — (DOCX) [file pone.0223877.s004.docx]

**S4 Table – Differential Scanning Fluorimetry and Dynamic Light Scattering results**

|  | **DSF** | | | **DLS** | | | | |
| --- | --- | --- | --- | --- | --- | --- | --- | --- |
| **Sample** | ***T*_m_**  **(°C)** | **C.I. ^a^**  **(°C)** | **N.M.^b^** | ***T*_aggr_**  **( °C)** | **C.I. ^a^**  **(°C)** | **N.M.^b^** | ***R_h_* ^c^**  **(nm)** | **Pd ^d^**  **(%)** |
| _His4-_K4 | 89,2^e^ | 88,6 – 89,7 | 2 | NP^f^ | | 2 | 4.2 | 16.0 |
| _His4-_K4/K3-_FLAG_ | 59,6 | 59,4 – 59,8 | 3 | 61,3 | 61,1 – 61,4 | 3 | 5.7 | 25.7 |
| K1-_His4_/K1-_FLAG_ | NP^f^ | | 3 | NP^f^ | | 3 | 5.2 | 29.0 |
| K2-_His4_/K2-_FLAG_ | 62,3^e^ | 61,7 – 62,8 | 3 | 80,6 | 80,5 – 80,7 | 4 | 5.7 | 27.2 |
| K1-_His4_/K2-_FLAG_ | 58, 7 | 58,5 – 58,9 | 3 | 71,3 | 71,2 – 71,4 | 3 | 5.7 | 31.5 |

^a^ 95% confidence interval.

^b^ Number of measurements.

^c^ Hydrodynamic radius

^d^ Polydispersity

^e^ Calculated from a low fluorescence signal event.

^f^ No measurable transition observed.
