# Supplementary material for: Occurrence and stability of hetero-hexamer associations formed by β-carboxysome CcmK shell components
Source: PLoS One. 2019 Oct 11;14(10):e0223877. doi: 10.1371/journal.pone.0223877 (PMC6788708; doi:10.1371/journal.pone.0223877)
Supplement: S1 List — (DOCX) [file pone.0223877.s005.docx]

**Preparation of pET26b-based vector for CcmK coexpression studies:**

The sequence indicated contains 4 cassettes flanked by T7 promoter/terminator sequences and containing RBS sequences.

Flanking SacI/KpnI sites permit integration in pBlueScript II SK+, for subsequent DNA manipulations. BglII and BlpI sites (underlined-italics) served in the final transfer to pET26b.Other indicated sites permit the insertion of CcmK sequences carrying different tags at either N or C-ter. They are shown following the next order on the sequence: SwaI, BamHI, PacI, AgeI, MfeI, SalI, BsrGI, HindIII.

GAGCTCTAA*AGATCT*CGATCCCGCGAAATTAATACGACTCACTATAGGGGAATTGTGAGCGGATAACAATTCCCCTCTAGAAATAAGATTTAAATACTTTAAGAAGGAGATATACC**ATG**GCACATCACCATCATGCTAGCGGCGAAAATCTGTACTTCCAGGGTGCCATGGCAATCGAGAGTGCGGCCGCACATCACCATCATTGATAGGATCCACTTCTCGAGTTAACTCGTGAGCAATAACTAGCATAACCCCTTGGGGCCTCTAAACGGGTCTTGAGGGGTTTTTTGCTGAAAGTACACGGCCGCATAATCGAAATTAATACGACTCACTATAGGGGAATTGTGAGCGGATAACAATTCCCCTCTAGAGTTAATTAAGTAAGTATAAGAAGGAGATATACC**ATG**GCAAGCTGGAGCCACCCGCAGTTCGAAAAGGGTGCTAGCGGCGAAAATCTGTACTTCCAGGGTGCCATGGCAATCGCTGTGTTCCAGAGTGCGGCCGCATGGAGCCACCCGCAGTTCGAAAAGTGACTAGTTACCGGTCACCTCTCGAGAGCAATAACTAGCATAACCCCTTGGGGCCTCTAAACGGGTCTTGAGGGGTTTTTTGCTGAAAGTACACTCCAGCATTACGAAATTAATACGACTCACTATAGGGGAATTGTGAGCGGATAACAATTCCCCTCTAGAAATAATTTTACAATTGTTTAAGAAGGAGATATACC**ATG**GATTACAAAGATGACGATGATAAGGCTAGCGGCGAAAATCTGTACTTCCAGGGTGCCATGGCACAAGCGGTGGAGTGCGGCCGCAGATTACAAAGATGACGATGATAAGTGACTAGTATGTCGACTCCTAGGACTCGAGCAATAACTAGCATAACCCCTTGGGGCCTCTAAACGGGTCTTGAGGGGTTTTTTGCTGAAACGATCCCGCGAAATTAATACGACTCACTATAGGGGAATTGTGAGCGGATAACAATTCCCCTCTAGAAATAATGTACATTAACTTTAAGAAGGAGATATACC**ATG**GCATACCCGTACGATGTTCCGGATTACGCTAGCGGCGAAAATCTGTACTTCCAGGGTGCCATGGCAGCCCACCAGAGTGCGGCCGCATACCCGTACGATGTTCCGGATTACGCATGACCTAGGAAAGCTTTCTCGAGA*GCTGAGC*AATAACTAGCATAACCCCTTGGGGCCTCTAAACGGGTCTTGAGGGGTTTTTTGGTACCAATTC

**Sequences of INDIVIDUAL GENES**

Underlined: Flanking XbaI/XhoI sites are for transfer to PET15b and analysis of expression/solubility of individual protein constructs.

Underlined for His4-, StrepTag-, FLAG- and HA-tagged sequences are SwaI/BamHI, PacI/AgeI, MunI/SpeI and BsrGI/HindiIII sites required for integration at 1^st^ to 4^th^ cassette of the pET26-based vector for coexpression studies, respectively (highlighted above with cyan and yellow background colors).

Construct start and stop codons are indicated in blue bold, black bold letters being used for Ccm start codon within N-ter tagged sequences.

Purification/labeling tags are shown in red, TEV-cleavage sequence in green.

> untagged CcmK1 Syn6803

cctctagAAATAAGATTTAAATACTTTAAGAAGGAGATATACc**atg**GCAATCGCTGTAGGTATGATCGAAACTCTGGGGT

TTCCGGCTGTTGTGGAAGCAGCCGATAGCATGGTAAAAGCGGCGCGCGTGACCTTAGTGGGCTATGAAAAGATTGGCAGC

GGTCGTGTCACCGTTATTGTTCGCGGGGATGTCAGCGAGGTGCAAGCGTCAGTGACGGCGGGTATCGAAAATATCCGTCG

TGTAAACGGTGGAGAAGTACTGTCAAACCATATCATCGCACGCCCACATGAAAATCTGGAGTATGTTTTACCGATTCGCT

ATACGGAAGCTGTGGAGCAGTTTCGTGAGATTGTAAACCCAAGCATCATCCGCCGTGCT**TAA**GATGTTTACTCCCGGGTA

GCGGCGAAAATCTGTACTTCCAGAGTGCGGCCGCACATCACCATCATTGATAGGATCCACTTCtcgaggat

> His4_TEV_CcmK1 Syn6803

cctctagAAATAAGATTTAAATACTTTAAGAAGGAGATATACC**ATG**GCACATCACCATCATGctagCGGCGAAAATCTGT

ACTTCCAGGGTGCC**ATG**GCAATCGCTGTAGGTATGATCGAAACTCTGGGGTTTCCGGCTGTTGTGGAAGCAGCCGATAGC

ATGGTAAAAGCGGCGCGCGTGACCTTAGTGGGCTATGAAAAGATTGGCAGCGGTCGTGTCACCGTTATTGTTCGCGGGGA

TGTCAGCGAGGTGCAAGCGTCAGTGACGGCGGGTATCGAAAATATCCGTCGTGTAAACGGTGGAGAAGTACTGTCAAACC

ATATCATCGCACGCCCACATGAAAATCTGGAGTATGTTTTACCGATTCGCTATACGGAAGCTGTGGAGCAGTTTCGTGAG

ATTGTAAACCCAAGCATCATCCGCCGTGCT**TAA**GATGTTTACTCCCGGGTAGCGGCGAAAATCTGTACTTCCAGAGTGCg

gccGCACATCACCATCATTGATAGGATCCACTTCtcgaggat

> Strep_TEV_CcmK1 Syn6803

cctctagAGTTAATTAAGTAAGTATAAGAAGGAGATATACC**ATG**GCAAGCTGGAGCCACCCGCAGTTCGAAAAGGGTGct

agCGGCGAAAATCTGTACTTCCAGGGTGCC**ATG**GCAATCGCTGTAGGTATGATCGAAACTCTGGGGTTTCCGGCTGTTGT

GGAAGCAGCCGATAGCATGGTAAAAGCGGCGCGCGTGACCTTAGTGGGCTATGAAAAGATTGGCAGCGGTCGTGTCACCG

TTATTGTTCGCGGGGATGTCAGCGAGGTGCAAGCGTCAGTGACGGCGGGTATCGAAAATATCCGTCGTGTAAACGGTGGA

GAAGTACTGTCAAACCATATCATCGCACGCCCACATGAAAATCTGGAGTATGTTTTACCGATTCGCTATACGGAAGCTGT

GGAGCAGTTTCGTGAGATTGTAAACCCAAGCATCATCCGCCGTGCT**TAA**GATGTTTACTCCCGGGTAGCGGCGAAAATCT

GTACTTCCAGAGTGCggccGCATGGAGCCACCCGCAGTTCGAAAAGTGACTAGTTACCGGTCACCTCtcgaggat

> FLAG_TEV_CcmK1 Syn6803

cctctagAAATAATTTTACAATTGTTTAAGAAGGAGATATACC**ATG**GATTACAAAGATGACGATGATAAGGctagCGGCG

AAAATCTGTACTTCCAGGGTGCC**ATG**GCAATCGCTGTAGGTATGATCGAAACTCTGGGGTTTCCGGCTGTTGTGGAAGCA

GCCGATAGCATGGTAAAAGCGGCGCGCGTGACCTTAGTGGGCTATGAAAAGATTGGCAGCGGTCGTGTCACCGTTATTGT

TCGCGGGGATGTCAGCGAGGTGCAAGCGTCAGTGACGGCGGGTATCGAAAATATCCGTCGTGTAAACGGTGGAGAAGTAC

TGTCAAACCATATCATCGCACGCCCACATGAAAATCTGGAGTATGTTTTACCGATTCGCTATACGGAAGCTGTGGAGCAG

TTTCGTGAGATTGTAAACCCAAGCATCATCCGCCGTGCT**TAA**GATGTTTACTCCCGGGTAGCGGCGAAAATCTGTACTTC

CAGAGTGCggccGCAGATTACAAAGATGACGATGATAAGTGACTAGTATGTCGACTCCTAGGACtcgaggat

> HA_TEV_CcmK1 Syn6803

cctctagAAATAATGTACATTAACTTTAAGAAGGAGATATACC**ATG**GCATACCCGTACGATGTTCCGGATTACGctagCG

GCGAAAATCTGTACTTCCAGGGTGCC**ATG**GCAATCGCTGTAGGTATGATCGAAACTCTGGGGTTTCCGGCTGTTGTGGAA

GCAGCCGATAGCATGGTAAAAGCGGCGCGCGTGACCTTAGTGGGCTATGAAAAGATTGGCAGCGGTCGTGTCACCGTTAT

TGTTCGCGGGGATGTCAGCGAGGTGCAAGCGTCAGTGACGGCGGGTATCGAAAATATCCGTCGTGTAAACGGTGGAGAAG

TACTGTCAAACCATATCATCGCACGCCCACATGAAAATCTGGAGTATGTTTTACCGATTCGCTATACGGAAGCTGTGGAG

CAGTTTCGTGAGATTGTAAACCCAAGCATCATCCGCCGTGCT**TAA**GATGTTTACTCCCGGGTAGCGGCGAAAATCTGTAC

TTCCAGAGTGCggccGCATACCCGTACGATGTTCCGGATTACGCATGACCTAGGAAAGCTTTCtcgaggat

> CcmK1_TEV_His4 Syn6803

CCTCTAGAAATAAGATTTAAATACTTTAAGAAGGAGATATACc**atg**GCAATCGCTGTAGGTATGATCGAAACTCTGGGGT

TTCCGGCTGTTGTGGAAGCAGCCGATAGCATGGTAAAAGCGGCGCGCGTGACCTTAGTGGGCTATGAAAAGATTGGCAGC

GGTCGTGTCACCGTTATTGTTCGCGGGGATGTCAGCGAGGTGCAAGCGTCAGTGACGGCGGGTATCGAAAATATCCGTCG

TGTAAACGGTGGAGAAGTACTGTCAAACCATATCATCGCACGCCCACATGAAAATCTGGAGTATGTTTTACCGATTCGCT

ATACGGAAGCTGTGGAGCAGTTTCGTGAGATTGTAAACCCAAGCATCATCCGCCGTGCGGGTAGCGGCGAAAATCTGTAC

TTCCAGAGTGCggccGCACATCACCATCAT**TGA**TAGgatcccggTCACCTCTCGAGAGCA

> CcmK1_TEV_Strep Syn6803

cctctagAGTTAATTAAGTAAGTATAAGAAGGAGATATACc**atg**GCAATCGCTGTAGGTATGATCGAAACTCTGGGGTTT

CCGGCTGTTGTGGAAGCAGCCGATAGCATGGTAAAAGCGGCGCGCGTGACCTTAGTGGGCTATGAAAAGATTGGCAGCGG

TCGTGTCACCGTTATTGTTCGCGGGGATGTCAGCGAGGTGCAAGCGTCAGTGACGGCGGGTATCGAAAATATCCGTCGTG

TAAACGGTGGAGAAGTACTGTCAAACCATATCATCGCACGCCCACATGAAAATCTGGAGTATGTTTTACCGATTCGCTAT

ACGGAAGCTGTGGAGCAGTTTCGTGAGATTGTAAACCCAAGCATCATCCGCCGTGCGGGTAGCGGCGAAAATCTGTACTT

CCAGAGTGCggccGCATGGAGCCACCCGCAGTTCGAAAAG**TGA**CTAGTTACCGGTCACCTCtcgaggat

> CcmK1_TEV_FLAG Syn6803

cctctagAAATAATTTTACAATTGTTTAAGAAGGAGATATACc**atg**GCAATCGCTGTAGGTATGATCGAAACTCTGGGGT

TTCCGGCTGTTGTGGAAGCAGCCGATAGCATGGTAAAAGCGGCGCGCGTGACCTTAGTGGGCTATGAAAAGATTGGCAGC

GGTCGTGTCACCGTTATTGTTCGCGGGGATGTCAGCGAGGTGCAAGCGTCAGTGACGGCGGGTATCGAAAATATCCGTCG

TGTAAACGGTGGAGAAGTACTGTCAAACCATATCATCGCACGCCCACATGAAAATCTGGAGTATGTTTTACCGATTCGCT

ATACGGAAGCTGTGGAGCAGTTTCGTGAGATTGTAAACCCAAGCATCATCCGCCGTGCGGGTAGCGGCGAAAATCTGTAC

TTCCAGAGTGCggccGCAGATTACAAAGATGACGATGATAAG**TGA**CTAGTATGTCGACTCCTAGGACtcgaggat

> CcmK1_TEV_HA Syn6803

cctctagAAATAATGTACATTAACTTTAAGAAGGAGATATACc**atg**GCAATCGCTGTAGGTATGATCGAAACTCTGGGGT

TTCCGGCTGTTGTGGAAGCAGCCGATAGCATGGTAAAAGCGGCGCGCGTGACCTTAGTGGGCTATGAAAAGATTGGCAGC

GGTCGTGTCACCGTTATTGTTCGCGGGGATGTCAGCGAGGTGCAAGCGTCAGTGACGGCGGGTATCGAAAATATCCGTCG

TGTAAACGGTGGAGAAGTACTGTCAAACCATATCATCGCACGCCCACATGAAAATCTGGAGTATGTTTTACCGATTCGCT

ATACGGAAGCTGTGGAGCAGTTTCGTGAGATTGTAAACCCAAGCATCATCCGCCGTGCGGGTAGCGGCGAAAATCTGTAC

TTCCAGAGTGCggccGCATACCCGTACGATGTTCCGGATTACGCA**TGA**CCTAGGAAAGCTTTCtcgaggat

All other CcmK Syn6803 sequences were mounted on the same way, just replacing CcmK1 above (blocks in grey above) by next DNA sequences:

> For CcmK2 Syn6803 constructs

GCAATCGCTGTGGGTATGATCGAAACACGCGGGTTTCCAGCGGTTGTGGAGGCGGCGGATTCAATGGTAAAAGCAGCGCGCGTTACCTTAGTGGGCTATGAAAAGATTGGCAGCGGTCGTGTAACCGTTATTGTGCGTGGGGATGTTAGCGAAGTCCAGGCAAGCGTCAGCGCCGGCATCGAGGCGGCAAATCGTGTGAATGGTGGGGAAGTACTGTCAACGCATATCATCGCACGCCCACATGAAAATCTGGAGTATGTTTTACCGATCCGTTATACCGAAGAAGTTGAACAGTTCCGTACGTACG

> For CcmK3 Syn6803 constructs

GCACAAGCGGTGGGAGTGATTCAAACCTTGGGCTTTCCGAGCGTGTTAGCGGCGGCGGATGCGATGCTAAAAGGGGGCCGGGTGACGCTGGTGTATTATGACCTGGCTGAACGAGGCAACTTTGTAGTAGCAATCCGAGGTCCCGTATCAGAGGTTAACCTTTCGATGAAGATGGGATTAGCAGCGGTAAACGAGTCCGTCATGGGAGGTGAAATCGTTAGCCATTATATTGTGCCGAACCCGCCCGAAAATGTGCTGGCGGTTCTGCCAGTGGAGTATACCGAAAAGGTTGCTCGTTTCCGGA

> For CcmK4 Syn6803 constructs

GCAGCCCAGAGCGCCGTGGGCAGCATTGAAACCATTGGCTTTCCGGGCATTCTTGCCGCCGCGGATGCGATGGTAAAAGCTGGTCGCATTACCATTGTGGGCTATATTCGTGCGGGCTCTGCGCGCTTTACGCTGAACATTCGTGGGGATGTGCAGGAAGTTAAAACGGCGATGGCTGCGGGCATCGATGCCATCAACCGTACAGAAGGAGCCGATGTGAAAACCTGGGTCATTATTCCGCGCCCACATGAAAATGTCGTTGCGGTTCTGCCGATCGATTTTAGCCCTGAAGTAGAACCCTTTCGCGAAGCAGCGGAGGGCCTGAACCGTCGCG

All other individual cases or combinations implying CcmK from Syn7942 were mounted on the same way, just replacing in the corresponding construct (first or second cassette) the sequence spreading from the start to the stop codons (bold blue font).

> CcmK3_His6 Syn7942 constructs

**ATG**CCAATCGCAGTGGGAACCATCCAAACCTTGGGCTTCCCGCCCATCATCGCAGCGGCCGATGCAATGGTAAAAGCCGC

GCGGGTGACGATCACGCAGTACGGCCTCGCGGAGAGCGCACAGTTCTTTGTCAGCGTACGTGGGCCGGTCAGCGAGGTGG

AGACCGCAGTTGAGGCGGGGCTGAAAGCGGTGGCGGAGACCGAGGGCGCCGAATTGATTAACTATATTGTGATCCCAAAC

CCACAGGAGAACGTGGAAACTGTGATGCCCATTGACTTCACGGCAGAATCAGAACCCTTTCGAAGCGGTAGTGCggccGC

AGGTAGTGGCGGTGCACATCACCATCACCATCAT**TGA**

> CcmK3_FLAG Syn7942 constructs

**ATG**CCAATCGCAGTGGGAACCATCCAAACCTTGGGCTTCCCGCCCATCATCGCAGCGGCCGATGCAATGGTAAAAGCCGC

GCGGGTGACGATCACGCAGTACGGCCTCGCGGAGAGCGCACAGTTCTTTGTCAGCGTACGTGGGCCGGTCAGCGAGGTGG

AGACCGCGGTGGAGGCGGGGCTGAAGGCGGTGGCGGAGACCGAGGGCGCCGAATTGATTAACTATATTGTGATCCCAAAC

CCACAGGAGAACGTGGAAACTGTGATGCCCATTGACTTCACGGCAGAATCAGAACCCTTTCGAAGCGGGAGTGCGGCCGC

AGGTAGTGGCGGTGCAGATTACAAAGATGACGATGATAAG**TGA**

> His6_CcmK4 Syn7942 constructs

**ATG**GCACATCACCATCACCATCATGctAGCGGCGGTTCTGGTGGCATGTCACAGCAAGCGATTGGCAGCCTGGAGACCAA

GGGCTTTCCGCCCATTCTCGCCGCCGCCGATGCAATGGTAAAAGCTGGTCGTATCACCATCGTGAGCTATATGCGGGCGG

GTAGCGCCCGCTTTGCGGTTAACATCCGGGGCGACGTGTCTGAAGTCAAAACTGCGATGGACGCGGGTATCGAAGCTGCT

AAAAATACCCCAGGTGGTACGCTGGAGACCTGGGTTATTATCCCGCGACCGCACGAAAATGTGGAAGCCGTGTTTCCAAT

CGGTTTTGGCCCAGAAGTAGAACAATACCGGCTGTCAGCGGAGGGCACTGGCTCGGGTCGTCGC**TGA**

> CcmK4_His6 Syn7942 constructs

**ATG**TCACAGCAAGCGATTGGCAGCCTGGAGACCAAGGGCTTTCCGCCCATTCTCGCCGCCGCCGATGCAATGGTAAAAGC

TGGTCGTATCACCATCGTGAGCTATATGCGGGCGGGTAGCGCCCGCTTTGCGGTTAACATCCGGGGCGACGTGTCTGAAG

TCAAAACTGCGATGGACGCGGGTATCGAAGCTGCTAAAAATACCCCAGGTGGTACGCTGGAGACCTGGGTTATTATCCCG

CGACCGCACGAAAATGTGGAAGCCGTGTTTCCAATCGGTTTTGGCCCAGAAGTAGAACAATACCGGCTGTCAGCGGAGGG

CACTGGCTCGGGTCGTCGCGGTAGTGCggccGCAGGTAGTGGCGGTGCACATCACCATCACCATCAT**TGA**

> CcmK4_FLAG Syn7942 constructs

**ATG**TCACAGCAAGCGATTGGCAGCCTGGAGACCAAGGGCTTTCCGCCCATTCTCGCCGCCGCCGATGCAATGGTAAAAGC

TGGTCGTATCACCATCGTGAGCTATATGCGGGCGGGTAGCGCCCGCTTTGCGGTTAACATCCGGGGCGACGTGTCTGAAG

TCAAAACTGCGATGGACGCGGGTATCGAAGCTGCTAAAAATACCCCAGGTGGTACGCTGGAGACCTGGGTTATTATCCCG

CGACCGCACGAAAATGTGGAAGCCGTGTTTCCAATCGGTTTTGGCCCAGAAGTAGAACAATACCGGCTGTCAGCGGAGGG

CACTGGCTCGGGTCGTCGCGGTAGTGCggccGCAGGTAGTGGCGGTGCAGATTACAAAGATGACGATGATAAG**TGA**
