# Supplementary material for: Occurrence and stability of hetero-hexamer associations formed by β-carboxysome CcmK shell components
Source: PLoS One. 2019 Oct 11;14(10):e0223877. doi: 10.1371/journal.pone.0223877 (PMC6788708; doi:10.1371/journal.pone.0223877)
Supplement: Attachment — Submitted filename: Response to Reviewers.docx [file pone.0223877.s013.docx]

Please find below in black letters our reply to reviewers comments. Other minor changes were introduced, mainly with the intention to improve the discussion. Most important are mentioned in the last portion of this letter, others can be easily visualized in the “Revised Manuscript with Track Changes” version.

**Reviewer #1:**

This manuscript provides compelling evidence for the specific association of ccmK1 with ccmK2, and ccmK3 with ccmK4. These paralogous carboxysome shell proteins are located in different regions of cyanobacterial genomes--cmmK1 and 2 are associated with genes encoding other carboxysomal components, while ccmK3 and 4 are colocated elsewhere.

Expressing these shell proteins in a variety of combinations, and purifying the hexamers, showed K1/K2 associations and K3/K4 associations.

Frustratingly, K3 could not be co-crystalized with K4, but the other experiments do seem to show that these two proteins do indeed associate.

My suggestions are relatively minor and follow below:

Line 28: replace ‘permitted to localize’ with ‘permitted the localization of’

It was replaced

Line 29-30: ‘Attempts to crystallize these CcmK3/K4 associations conducted to the unambiguous elucidation of a CcmK4 homo-hexamer structure’ needs to be rephrased

Replaced by “*The crystallization of these CcmK3/K4 associations conducted to the elucidation of a structure corresponding to the CcmK4 homo-hexamer*”. We hope it will be better.

Line 32: ‘in frame with’ should be replaced with ‘within the context of’
Line 66: ‘single protein paralogs’ Would rephrase as ‘single proteins’
Lines 95 and 96, and throughout the manuscript: double-check to be certain that taxon names are italicized
Line 107: ‘chimio’ should be replaced with ‘chemically’
Line 135: delete ‘for ulterior analyses’
Line 136: delete ‘steps’ after ‘3-4’
Line 143: replace ‘ulterior’ with ‘subsequent’
Line 163: delete ‘next’
Line 202: remove ‘equipment’
Line 210: remove ‘a’

All these proposed changes have been introduced.

Line 264: ‘permitting the production of soluble CcmK1 (K1) and CcmK2 (K2) with any of the 4 peptides’. I do not follow this.

We replaced the sentence by: “*Thus, CcmK1 (K1) and CcmK2 (K2) were expressed and remained soluble regardless of the peptide tag identity at C-terminus (Fig. S2)*” that we hope will be clearer.

Line 292: ‘Such bands were net for K1, K2 and K4 cases’…? Clarify?

We introduced two modifications: 1) we specified in legend to Fig. S3A the meaning of underlined combinations shown in the table, something that we forgot to mention in the submitted version; 2) in the main text now, it is written: “*Cases combining His_4_- and FLAG-tagged versions of the same paralog served as positive controls (underlined in table of Fig. S3A), providing an indication of signal level attained for homo-hexamers.* *Such bands were clearly detected for K1-His_4_/K1-FLAG, K2-His_4_/K2-FLAG, K4-His_4_/K4-FLAG and His_4_-K4 /K4-FLAG.*”

Line 386: replace ‘permitted to identify’ with ‘identified’
We did.

Fig. 3: The information schematized in Fig. S4A should probably be placed somehow in figure 3 to help the reader follow this

Figure 3 has been modified accordingly. Now, two panels are present and numerical correspondences between gene constructs and analyzed cases are given for better clarity. We hope it is now better and we apologize for complicating the reading of the first submitted version of the manuscript.

Fig 5C—only see one band. Should we see two, since the text is suggesting K3K4 interaction? Perhaps put more info in the text or figure legend to understand what we are seeing here?

Unfortunately, the two proteins present an almost identical electrophoretic mobility. This is now mentioned in the figure legend: “*Please notice that His_4_-CcmK4 and CcmK3-FLAG bands could not be resolved*.”

Line 463: ‘Besides’ should probably be replaced with ‘Furthermore’
Line 482: Replace ‘as indicate the WB’ with ‘as indicated by the WB’
Line 508: Replace ‘revealed’ with ‘was’
Line 514: Replace ‘to monitor’ with ‘the monitoring of’

All proposed changes have been introduced.

Fig. 6: use larger symbols so the traces can be distinguished
The figure has been modified accordingly. However, larger symbols imposed to show alternate data points (1 every 2), otherwise it became impossible to recognize each trace. This is mentioned in the legend.

We hope the modifications are helpful.

Line 546: insert ‘the opportunity’ after ‘permit’
Line 575: replace ‘non-rare’ with ‘many’

These changes were introduced in the revised version.

We sincerely thank the reviewer for helping us in ameliorating the readability of our study.

Reviewer #2: Garcia Alles et al. describe a series of experiments designed to elucidate interactions between different CcmK* homologs of Synechocystis. To their credit, the work is very thorough, with investigations of the influence of different tags and tag positions on subunit interactions, and experiments where all 4 proteins are simultaneously expressed. The initial conclusions from Western blotting are backed by mass spectrometry analysis. The CcmK1/CcmK2 complex and CcmK3/CcmK4 complex are also tested for their ability to pack laterally using AFM. Homology modelling and sequence comparisons are used to investigate the CcmK3/CcmK4 complex. The stability of this complex and ability of subunits to exchange is also tested. In general, this is a careful, very thorough study; the authors were unfortunate in that their major conclusion was anticipated by a competitor’s work, but this paper adds depth and nuance, and certainly deserves a place in PLOS One.

While the work seems technically sound, I do think the presentation could be improved. Phrasing is often awkward, sometimes to the point where the author’s intentions are not clear. The paper would greatly benefit from editorial attention from a native english speaker. The figures are also difficult to follow in terms of the experimental design and what a positive/negative readout tells you. I think the authors should consider adding some of the explanatory panels currently in the supplementary figures to the main figures. Fig. 3 in particular would benefit.

The reviewer is right. As indicated for reviewer 1, Figure 3 has now been modified. An explanatory panel was added and numerical correspondences between gene constructs and analyzed cases are given for better clarity.

Similarly, we modified figure S4 to make things clearer. The same table with numeric correspondences is presented again, and the panel B was split in two pieces to permit a clearer attribution of data collected for each analyzed case 1 to 8.

Similarly, the table in Fig. S3 and labeling of Fig. 7 have been modified with the intention to make attribution of studied combinations to corresponding data clearer.

We hope the manuscript is now easier to follow. We apologize for complicating unnecessarily the reading of the first submitted version of the manuscript.

The discussion is overly long (seven pages) and rambling. It reiterates many of the points made in the introduction and results at some length. It also introduces a great deal of fairly speculative ideas. I think the paper would be much more readable if the authors tried to limit this, focussing on the ideas that they think most likely, or a handful of their best supported speculative ideas.

We agree the discussion is long. We tried to rearrange some paragraphs and to delete unnecessary portions. However, it was necessary to update the discussion in the light of very recently published data that demonstrate the presence of CcmK3 and CcmK4 in carboxysome shells (two new citations were introduced). In consequence, the text shrank only slightly, passing from 17715 characters to 15190 characters now (15% shorter).

I admit the discussion is still long, but I think it is necessary. We dedicate the first 2,5 pages to summarize and put together the new observations. The next 1,5 pages are necessary to recapitulate published data on CcmK3/CcmK4 knockouts, and to discuss our data within the frame of the proposed shell-capping scenario, which can be considered speculative. One page more is written to discuss two other possible functions that emerge on the light of our data, which we believe deserve consideration, in spite of the fact that they are speculative too. The last page intends to present perspectives for future experimentation.

We hope the reviewer will find the new version clearer, less repetitive.

The section on sequence and structural arguments for CcmK3 forming a complex with CcmK4 is based largely on arguments from homology models and molecular dynamics, which have fairly strong inherent biases towards the starting model. I feel this section could again be shortened.

We agree on the need to be cautious when considering these data, and that is why we intentionally wrote the paragraph in a conditional style. However, we think that 3D homology models and MD simulations still provide some clues on why CcmK3 might be more stable when embedded within CcmK4 monomers than alone. This could be of help for other investigations, for instance for crystallography attempts, or to compare the solution behavior of CcmK3 from different organisms.

Figure 1 – I am not opposed to the idea of using lines to guide the eye, but perhaps the vertical lines could be less prominent where a single gel is used, in order to not obscure where adjacent lanes are from the same gel. E.g. a less thick, and perhaps dashed line would serve equally well for demarcation.
White vertical lines have been introduced in Fig. 1 to highlight gel discontinuities in the mounted figure. We hope this is what reviewer asked for.

637 – lack of evidence from coevolutionary algorithms may simply reflect that there are not enough distinct beta-cyanobacterial sequences.

The reviewer is correct. We deleted in the revised version the sentence, to avoid unnecessary speculation.

The legend for Fig 3 does not really allow the experiment to be understood as presented. For example, on what basis is each lane labelled with the black and purple letters at the top? What is the difference between lanes? Are all tags actually present in each experiment? I understand the conclusion the authors reach, and can see evidence for individual interactions, but don’t understand how each experiment was set up, or how to interpret the absence of WB signal with each of the tags being interrogated.

As indicated above, Figure 3 has now been modified accordingly. An explanatory panel was added and numerical correspondences between gene constructs and analyzed cases are given for better clarity.

We hope it is now clearer. We apologize for the inconvenience while reading the first submitted version of the manuscript.

Fig 4B again lacks context. What model is this? One of the homology models, or a post MD model? The orientation of the image is also not immediately clear to the reader. Picking standard orientation (concave or convex surface) and perhaps using surface colour to show how these residues protrude might help.

In original Figure 4B legend was written: “*Presumed localization on the Syn6803 CcmK3_1 modeled structure of some notable residue substitutions*”. The CcmK3_1 model is the one prepared with SWISS-MODEL, as indicated in the main text, 415-429 : “*To further visualize these differences, homology models were built using SWISS-MODEL (CcmK3_1) and PHYRE2 (CcmK3_2) algorithms. The resulting monomer models were similar…*”. We therefore do not understand very the first question. However, to further clarify the text, we have replaced the legend sentence by “*Presumed localization of some notable residue substitutions in the CcmK3 Syn6803 structure furnished by SWISS-MODEL (CcmK3_1 in the main text), when compared to other CcmK*”

As far as the orientation problem and surface colors is concerned, we agree with the reviewer the images were not clear enough. We modified the two panels of the figure, and the corresponding legend. We hope things are now more easy to follow.

Fig S3 – what does underlining indicate in the table?

 We apologize, we forgot to mention it. The legend now reads: “*Studied cases are listed in the table. In bold and underlined are the combinations of the same paralog expected to produce doubly-tagged homo-hexamers (positive controls)”.*

We thank the reviewer for calling our attention on this detail.

OTHER NOTABLE MODIFICATIONS:

517-520: We removed the sentence in bold below, which was unjustified. In consequence, we rearranged a bit the text as follows:

First submitted:” *In comparison, the K1 homo-hexamer seemed reluctant to denaturation, fluorescence readings changing slowly and weakly with temperature, which prevented the determination of a T_m_ value. In the case of K4 and K2, T_m_ of 89.2 and 62.3 °C were measured, respectively.* ***However, especially for K2, signal amplitudes were low, suggesting that such event could be related to the disruption of dodecamer structures rather than to hexamer unfolding*.**”

Revised version:” *In comparison, T_m_ of 89.2 and 62.3 °C were measured for K4 and K2, respectively, whereas fluorescence readings with the K1 homo-hexamer changed slowly and weakly with temperature, which prevented the determination of a T_m_ value*.”

Discussion, 651-658:

The next portion of text was added to take into consideration freshly published data that demonstrate that CcmK3 and CcmK4 (YFP-labeled) can be detected in Syn7942 carboxysomes. Since only CcmK4 had been detected by MS in another study from the same group, we discuss it as possibly supporting a shell-capping model:

“*Also in agreement with a peripheral role of K3/K4 complexes, the two YFP-labeled proteins could be visualized in Syn7942 cells [50], whereas only CcmK4 was detected by MS when wild-type carboxysomes were purified [51]. This suggests that peripheral K3/K4, but not shell integrated K4 homo-hexamers, might have been lost during carboxysome isolation. Unfortunately, other known carboxysome components (e.g. CcmO, CcmN) were neither detected, making difficult to conclude on the real reasons behind the absence of CcmK3 detection. Investigating the fate of YFP-labeled K3 and K4 after purification of the corresponding fluorescent carboxysomes might permit to clarify this point*.”
